# Lovers, not fighters: docility influences reproductive fitness, but not survival, in male Cape ground squirrels, *Xerus inauris*

**DOI:** 10.1007/s00265-023-03421-8

**Published:** 2024-01-04

**Authors:** Miyako H. Warrington, Sienna Beaulieu, Riley Jellicoe, Sjoerd Vos, Nigel C. Bennett, Jane M. Waterman

**Affiliations:** 1 Department of Biological and Medical Sciences, Oxford Brookes University, Oxford, UK; 2 Department of Biological Sciences, University of Manitoba, Winnipeg, MB Canada; 3 Graduate School of Life Sciences, University of Utrecht, Utrecht, Netherlands; 4 Mammal Research Institute, Department of Zoology and Entomology, University of Pretoria, Pretoria, 0002 South Africa

**Keywords:** Alternative reproductive tactic, Lifetime fitness, Male groups, Paternity, Personality, Sciurid, Survival

## Abstract

Over their lifetime, individuals may use different behavioural strategies to maximize their fitness. Some behavioural traits may be consistent among individuals over time (i.e., ‘personality’ traits) resulting in an individual behavioural phenotype with different associated costs and benefits. Understanding how behavioural traits are linked to lifetime fitness requires tracking individuals over their lifetime. Here, we leverage a long-term study on a multi-year living species (maximum lifespan ~ 10 years) to examine how docility (an individual’s reaction to trapping and handling) may contribute to how males are able to maximize their lifetime fitness. Cape ground squirrels are burrowing mammals that live in social groups, and although males lack physical aggression and territoriality, they vary in docility. Males face high predation risk and high reproductive competition and employ either of two reproductive tactics (‘natal’ or ‘band’) which are not associated with different docility personalities. We found that although more docile individuals sired more offspring on an annual basis, docility did not affect an individual’s long-term (lifetime) reproductive output. Survival was not associated with docility or body condition, but annual survival was influenced by rainfall. Our findings suggest that although docility may represent a behavioural strategy to maximize fitness by possibly playing a role in female-male associations or female mate-choice, variations in docility within our study population is likely maintained by other environmental drivers. However, individual variations in behaviours may still contribute as part of the ‘tool kit’ individuals use to maximize their lifetime fitness.

**Supplementary Information:**

The online version contains supplementary material available at 10.1007/s00265-023-03421-8.

## Significance

Male grouping is rare in animal societies, and represents a form of cooperation and social tolerance among males who are typically intolerant of one another and compete aggressively for reproductive access to females. However, Cape ground squirrel males lack physical aggression and live all year-round in either mixed-sex or all-male groups. We examined the association between docility and reproductive success and survival to investigate whether docility is beneficial to males. We found that more docile males sired more offspring on an annual basis, but docility was unrelated to lifetime reproductive success and survival. These findings indicate that docility may represent a behavioural strategy used to maximize reproductive success in a group-living ground squirrel. However, the benefit of docility variation is nuanced in this species.

**Supplementary Information:**

The online version contains supplementary material available at 10.1007/s00265-023-03421-8.

## Introduction

Over their lifetime, individuals strive to maximize their fitness. Individuals may vary in their investment into somatic maintenance and reproduction (Williams 1966; Stearns 1992), with each individual having distinct constraints related to their physical, physiological, or behavioural phenotype. These constraints affect the strategies an individual could employ. Thus, different individuals may employ different strategies to manage their life histories or maximize their fitness (Biro and Stamps 2008). Behavioural strategies may include consistent behavioural differences among individuals (hereafter, ‘personality’), resulting in an individual behavioural phenotype (Sih and Bell 2008). As personality may affect survival and fitness (Biro and Stamps 2008; Smith and Blumstein 2008), individuals with differing personalities may manage their reproductive efforts differently. Thus, personality types may be part of the ‘tool kit’ that individuals use to maximize their lifetime fitness.

An individual’s personality type interacts with external factors such as environmental conditions or the social environment, which may have fitness consequences (Biro and Stamps 2008). In social animals, behavioural interactions between conspecifics (hereafter, ‘social behaviour’; Alexander 1974) may influence future interactions (or lack thereof) between individuals, and thus the costs and/or benefits associated with these social associations. For example, in female eastern grey kangaroos, *Macropus giganteus*, shy individuals foraged in larger group sizes (Best et al. 2015), which may confer benefits associated with reduced predation risk in larger herds (Carter et al. 2010). In fact, the extent that an individual tolerates the proximity of other individuals (hereafter, ‘social tolerance’, DeTroy et al. 2022) and their tendency to associate with other individuals (hereafter, ‘sociability’; Gartland et al. 2021), may have consequences to individual fitness (Gartland et al. 2021; DeTroy et al. 2022). Consequently, social tolerance and sociability may lead to diverse social associations and interactions (Kappeler et al. 2019; Gartland et al. 2021), which may have downstream consequences for how individuals are able to manage their fitness.

To investigate the influence of personality types in animals, one must obtain repeated behavioural measures of particular individuals over time. This is often achieved by capturing (trapping) and handling animals. The animal’s behavioural response to these respective events may be scored categorically, yielding a measure of docility. Docile individuals are generally quiet, easy to manipulate, and do not struggle while being handled (Réale et al. 2007). However, it remains challenging to determine how docility is related to the way animals interact with each other (instead of to a human being). Docility may be associated with aggression directed at predators (hereafter, ‘defensive aggression’) or at conspecifics (hereafter, ‘social aggression’; terms from Blumstein et al. 2013). For example, less docile animals were more aggressive in mirror-image experiments, a behavioural assay that assumes that attacking a mirror image represents attacking a conspecific (e.g., Boon et al. 2007; Haigh et al. 2017). Docility has also been associated with high social tolerance (Plusquellec and Bouissou 2001), such that increased docility may allow for, or enhance, social interactions and/or social living benefits. Alternatively, docility may be related to behavioural traits that are not necessarily associated with social behaviours, that nonetheless may affect lifetime fitness. Docility may be related to boldness (Réale et al. 2007), activity level and exploration (Martin and Réale 2008; Ferrari et al. 2013), and reactive inclinations (e.g., proactive or reactive tendencies; Martin and Réale 2008). Thus, individual variations in docility may be related to how individuals manage their life histories and their lifetime fitness.

Understanding how behavioural traits are linked to lifetime fitness requires the tracking of individuals over their lifetime. Species that survive multiple breeding seasons allow for the examination of behavioural variation (i.e., consistency, plasticity) over an individual’s lifetime, and the role this behavioural variation may play in an individual’s lifetime fitness. Here, we leverage a long-term study (20 + years) on a multi-year living species (lifespan ca. 9–11 years, Weigl 2005; Warrington et al. 2022) to examine how a behavioural measure may contribute to how males are able to maximize their lifetime fitness.

The Cape ground squirrel (*Xerus inauris*) is a group-living sciurid (Skurski and Waterman 2005). Females live in matrilineal family groups and adult males either delay dispersal and remain in their natal groups (hereafter, ‘natal’ males), or may disperse and join other dispersed males in roving all-male bands (hereafter, ‘band’ males). These two grouping types represent two discrete reproductive tactics (Scantlebury et al. 2008). Consequently, both group types are associated with a distinct social structure; natal groups are characterized by stable group memberships, while all-male bands are characterized by dynamic daily membership (Waterman 1995), referred to as a fission-fusion system (Aureli et al. 2008). Despite the differences between natal groups and all-male bands, both group types are associated with antipredator benefits, such as decreased individual vigilance in larger groups (Waterman 1997; Edwards and Waterman 2011). Consequently, male grouping following dispersal has been attributed to an individual’s strategy to minimize predation risk (Waterman 1997).

Both natal and band males have on average equal annual reproductive success (Manjerovic and Waterman 2015), however, males face high competition for paternity. Female Cape ground squirrel oestrus is highly asynchronous and unpredictable, with an operational sex ratio on the day of oestrus of 11 males: 1 female (Waterman 1996, 1998, 2010; Manjerovic et al. 2022). A female will mate with an average of four mates during her 3-h behavioural oestrus (Waterman 1996, 1998, 2010), and litter sizes are small (1–2 young; Waterman 1996). Thus, reproduction is highly skewed with approximately 1/3 of males siring offspring (Manjerovic and Waterman 2015; Manjerovic et al. 2022). Despite the high competition for limited paternity, male Cape ground squirrels lack physical aggression and territoriality (Waterman 1995, 1997), and natal males join band males with pre-copulatory competition limited to competitive searching on the day of oestrus (Waterman 1998). Males compete via post-copulatory sperm competition (Manjerovic et al. 2008) and have some of the largest testes amongst sciurids (Manjerovic et al. 2008). Currently, it remains unknown what drives the high reproductive skew in males of this species (Manjerovic and Waterman 2015); but perhaps other forms of individual variation, such as docility, may affect reproductive success.

Cape ground squirrels have been shown to have docility personality types (Warrington et al. 2022). As docility may be associated with traits that affect survival (e.g., antipredator defensive, aggression, Blumstein et al. 2013), traits that directly influence social interactions (e.g., social aggression/boldness; Best et al. 2015), or traits that indirectly influence social interactions by influencing rates/probabilities of encountering individuals (e.g., activity level and exploration; Ferrari et al. 2013), we broadly hypothesized that docility may also influence fitness in Cape ground squirrels. Following the methods of Réale et al. (2007), we quantified behavioural expressions of docility in males during trapping and handling and examined their association with (1) annual reproductive success, (2) lifetime reproductive success, (3) annual survival, and (4) on-site persistence (a proxy for lifespan). As behavioural traits may signal or indicate male quality, females may use behavioural traits to select mates (Schuett et al. 2010). If docility is related to traits that females may use for selection (e.g., social aggression, Qvarnström and Forsgren 1998), then we predict that docility would be related to reproductive success in males. If docility is related to traits that influence survival, such as traits that enhance the acquisition of social benefits (e.g., favourable social positions, Armitage and Van Vuren 2003), then we predict that docility would be associated with survival.

## Methods

### Study site

Animal trapping and morphological measurements have been collected as part of an ongoing long-term project on wild Cape ground squirrels at S.A. Lombard nature reserve (4600-ha), which is located 18 km northwest of Bloemhof, South Africa (27˚35’S, 25˚23’E). We used trapping data from May until August (austral winter) 2011–2021 to determine reproductive fitness and survival in individual squirrels. However, docility was only assessed from 2014 to 2019. It was not possible to record data blind because our study involved focal animals in the field.

The site habitat is a floodplain characterized by dry *Cymbopogon-Themeda* veld and black soil turf veld, with patches of bush and pan areas (Van Zyl 1965). In years of high rainfall, vegetation and seeds, which are food sources for Cape ground squirrels, are abundant (O’Brien et al. 2021; Manjerovic et al. 2022). On-site, natural predators of Cape ground squirrels include mammal, reptile and avian predators, such as black-backed jackals (C*anis mesomalas*), Cape cobra (*Naja nivea*), black-shouldered kites (*Elanus axillaris*), and pale chanting goshawks (*Melierax canorus*). Ground squirrel burrows are concentrated in several distinct areas of the site characterized by different levels of predation and human activity (Unck et al. 2009).

### Trapping and body measurements

From 2011 until 2021, we trapped 451 different males over 1805 trapping occasions. Throughout the field season, we performed daily trapping rounds (2–4 rounds/day; 70 traps/round) throughout the day (~ 08:00–17:30) with Tomahawk live traps (15 × 15 × 50 cm, Tomahawk Live Trap co., Tomahawk, WI, USA) baited with peanut butter and bird seed (Waterman 1995). To minimize heat stress, traps were fitted with shade covers and checked routinely at approximately 1-hour intervals. Squirrels were marked with a pit tag (AVID USA) for permanent identification, and for identification at a distance, a dorsal freeze mark (Freeze Spray, CRC Industries Inc., USA; Rood and Nellis 1980) and a black hair dye mark (Rodol D; Lowenstein and Sons Inc., New York, NY, USA). For each trapped male we: (1) measured body mass to the nearest 0.5 g using a spring scale (Pesola AG, Baar, Switzerland); (2) measured spine length from the base of the skull to the base of the tail, with a tape measure; (3) assessed reproductive condition – adult males are scrotal year-round and are easily distinguished from sub-adults who are either non-scrotal or partly scrotal; (4) collected 1–3 mm of skin from the tail tip of each individual to use for subsequent parentage analysis; and, (5) scored docility, defined as an animal’s reaction to handling by humans (Réale et al. 2007). We released each individual back into the area in which they were caught.

We calculated body condition using principal component analysis (PCA) using the R package ‘psych’ (Revelle 2022) as in Tranquillo et al. (2022) using male body mass (g) and the spine length (mm) average for each male for all measurements taken that year. Body condition was then defined as the second component (second component loadings: 0.707 for body mass, -0.707 for spine length) as heavier males had a higher score than lighter males of the same spine length.

### Docility scoring

We assessed docility on 914 occasions for 274 males from 2014 to 2019 (year [N_trap_ = number of trapping occasions, N_unique_ = number of unique males]: 2014 [N_trap_ = 55, N_unique_ =24]; 2015 [N_trap_ = 227, N_unique_ = 80]; 2016 [N_trap_ = 83, N_unique_ =73]; 2017 [N_trap_ = 66, N_unique_ = 61]; 2018 [N_trap_ = 216, N_unique_ = 119]; 2019 [N_trap_ = 267, N_unique_ = 125]). Docility is defined as an individual’s reaction to trapping and handling, whereby docile individuals are generally quiet, easy to manipulate, and do not struggle while being handled (Réale et al. 2007). Docility has been associated with aggression in several species with more aggressive individuals struggling more during handling (Boon et al. 2007; Haigh et al. 2017; although see Blumstein et al. 2013).

We based our docility scoring on the methods of Réale et al. (2000) and assigned scores during four aspects of trapping and handling (Warrington et al. 2022): approach, transfer, handling, and release. Docility behaviours were distinct and easy to qualify. Scoring was as follows: (a) approach, the response of the subject during the handler’s approach to the trap was classified as: 0 - is quiet and still; 1 - starts alarm calling and hissing when the handler approaches within one meter of the trap; and 2 - reacts to the handler from > 1 m, alarm calling, hissing and thrashing; (b) transfer from the trap to the handling bag: 0 - runs into the bag without protest; 1 - resists, but enters the bag after 30 to 60 s, the handler may have to bang on the trap; 2 - strongly resists bagging, the handler must shake or reposition the trap or open the back and push the squirrel into the bag; (c) handling: 0 - quiet and still, no perceptible reaction; 1 -struggles, snorts, and alarm calls less than half the time, but handling is manageable; and 2 - struggles, snorts, alarm calls more than half the time, making handling very difficult; and (d) upon release, the subject 0 - walks away; 1 - runs away. Scores reflect the degree of reaction; high-scoring individuals were less docile, and low-scoring individuals were more docile. After handling each ground squirrel, we released individuals into the area in which they were captured. As different researchers took estimates on docility, we assured similarity of docility scoring by training inexperienced researchers with an experienced researcher for several weeks and provided each researcher/trapping kit with a detailed written docility scoring protocol. We also included the handler identity as a random effect in subsequent models.

### Reproductive success

Paternity assessments followed those of Manjerovic and Waterman (2015). DNA was extracted from tail skin tissue collected in the field during trapping using a DNeasy Kit (Qiagen Inc., Valencia, California). We used 19 species-specific microsatellite loci to genotype all individuals (Shave and Waterman 2017) and determined the paternity of genotyped juveniles using CERVUS v.3.0 (Marshall et al. 1998; Kalinowski et al. 2007), which assigns parentage based on simulated population allele frequencies using a likelihood-based approach taking into account the proportion of the population sampled and the probability of mistyping errors. We only included individuals whose paternity could be definitively assigned with a confidence level greater than 95% (Manjerovic and Waterman 2015).

We examined two fitness variables: (1) annual fitness, the number of offspring a male sired in each year, and, (2) lifetime fitness, the number of offspring that a male sired over his lifetime. Males that were trapped in 2021, and hence potentially still breeding, were excluded from further lifetime fitness analyses.

### Survival

Survival estimates were determined by an individual’s trapping history. We quantified survival in two different ways. First, we determined annual survival, defined as whether the individual was sighted the following year. We attributed disappearances to death, which we assume to be the cause of the vast majority of disappearances, but we also note that we cannot determine the fate of all individuals that disappear as is a common challenge in small mammal studies (Murray and Patterson 2006). Second, we determined on-site persistence, the number of years that a male was trapped on site as an adult. On-site persistence often underestimates a band male’s lifespan because most males have dispersed into the area from outside the field site, and male dispersal age varies from 3 to 5 years of age (Waterman 1995; O’Brien et al. 2021). For natal males that were born on-site (n = 89/274), we cannot determine the fate of males that disappeared during the study period as they may have dispersed outside the study site. To account for this variation in on-site persistence estimates, we included reproductive strategy (‘band’ or ‘natal’) in all survival models.

### Reproductive tactic

For each male, we determined the reproductive tactic employed at the time of trapping, using methods as described in Warrington et al. (2022). We observed squirrels using 10 × 50 binoculars and 15-45 × 60 spotting scopes from observation towers or hides that were mounted on top of vehicles at a distance of 50 to 100 m (Scantlebury et al. 2008; O’Brien et al. 2021). We tracked males from (1) morning emergence, until they left to forage, and (2) from afternoon foraging when squirrels tended to be with their social group in the vicinity of their burrow cluster until evening immergence (Waterman 1995; Unck et al. 2009). Male reproductive tactic was determined by observing the location and social organization of sleeping groups, or the individuals’ trapping history. Since band males move over a larger home range compared to natal males (band males home range = approximately 31-ha; natal males = 11-ha; Manjerovic and Waterman 2015) and also sleep in different vacant burrows, while natal males return to the same burrow every night (Waterman 1995), male tactic can also be determined using detailed trapping information on within-season trapping location and sleeping locations (Warrington et al. 2022). Males with insufficient information to support a reproductive tactic assignment were thus excluded from further analyses.

### Statistical analysis

All data manipulation and statistical analysis were done in R version 4.3.0 (R Core Team 2023). We used R packages ‘tidyverse’ (Wickham et al. 2019) for code organization, ‘dplyr’ (Wickham et al. 2020) for data manipulation, ‘ggpubr’ for data visualization (Kassambara 2020), ‘rptR’ (Stoffel et al. 2017) for repeatability analysis, and ‘MCMCglmm’ (Hadfield 2010) and ‘coda’ (Plummer et al. 2006) for multivariate models and model diagnostics.

### Repeatability estimates

A previous study on this population found that docility during transfer and handling was repeatable (Warrington et al. 2022). However, to confirm repeatability of docility measures in this study, we examined consistent individual differences in trapping behaviours using generalized linear mixed models using the R package ‘rptR’ version 0.9.22 (Nakagawa and Schielzeth 2013). We ran four separate models for each docility response variable (docility during approach (M1), transfer (M2), handling (M3) and release (M4). For each model, we included the following fixed factors which were previously shown to significantly affect repeatability of docility behaviours (Warrington et al. 2022): (1) capture, whether the individual was trapped for the first time (first time = 1, all captures after the first = 0); (2) tenure, the length of time in years that the individual has been observed in the study population as an adult, and (3) rainfall, the total precipitation from July of the previous year until June of the sampling year, which represents the amount of rainfall prior to the austral winter season and is associated with plant productivity (Van Zyl 1965). We also included three random variables: (1) tag, the individual identity of the male squirrel; (2) area, in which area of the field site the individual was captured, and (3) handler ID, the human observer that captured the squirrel.

Tenure and rainfall were all z-standardized (mean = 0 and standard deviation = 1) prior to analysis. Confidence intervals were calculated around repeatability estimates using a non-parametric bootstrap with no permutation. We modelled approach, transfer and handling using a Poisson distribution (link = log), and release using a binomial distribution (link = logit). Repeatability estimates were considered significant based on confidence intervals with lower intervals > 0.1, and confirmed with *p*-values obtained from a likelihood ratio test (LRT).

### Effects of repeatable behaviours on reproductive fitness

As docility during transfer and handling were found to be repeatable, we fitted a multivariate GLMM using R package ‘MCMCglmm’ (Hadfield 2010) to investigate among-individual variance and covariance for fitness, reproductive tactic, transfer and handling. We included reproductive tactic as a covariate because relative fitness differences between different reproductive tactics may vary from year to year given that the body condition response to annual rainfall differs between band and natal males (O’Brien et al. 2021). We ran a separate model for each measure of reproductive fitness: (M1) annual offspring continuous, the number of offspring an individual had sired that breeding season; (M2) annual offspring binary, whether an individual sired any offspring that year, whereby zero offspring = 0, and ≥ one offspring = 1; (M3) total offspring continuous, the number of offspring sired since 2011 (start of study); and (M4) total offspring binary, whether any offspring were sired during the individual male’s lifetime, whereby zero offspring = 0 and ≥ one offspring = 1. For total offspring (M3 and M4) analyses, we excluded individuals that were captured in 2021 (and thus still breeding), and all individuals that were first captured as adults (as they may have sired offspring outside the study site, or sired offspring prior to the start of this study). In both situations, inclusion of these individuals may lead to under-estimating lifetime fitness. Hence, all males in M3 and M4 models have been tracked for their entire life from juvenile/subadult stage to adulthood. Docility during transfer and handling, and the continuous fitness measures were fitted as Poisson, and the binary fitness measures were fitted as categorical. Reproductive tactic, whereby natal = 0, and dispersed band males = 1, was also fitted as a categorical variable. We fitted the following fixed effects: capture (tested as a fixed effect for transfer and handling only), tenure, rainfall and body condition (z-centered to improve model fit). Tag, area and handler ID were fitted as random effects (Table 1).

We estimated within- and among-individual covariance by fitting an unstructured ‘us’ R-matrix (within-individual variation) for tag, and G-matrix (among-individual covariances). We used non-informative parametric-expanded Wishart priors throughout all models, and ran all models for 4 000 000 iterations, with a burn-in of 5000 and thinning interval of 2000. Successive samples from the posterior distribution had low autocorrelation (the majority was *r* < 0.02, while all were *r* < 0.05). To examine the correlation between response variables, we standardized model covariance response variables (handling, tactic, and fitness) to a scale from − 1 to 1 as described in Houslay and Wilson (2017a). Correlations were determined to be significant if the 95% confidence interval of the correlation excluded zero.

### Effects of repeatable behaviours on survival

We also examined among-individual variance and covariance for survival, transfer and handling, using the same methods as described above. We also included reproductive tactic as a covariate because survival may differ between males of different reproductive tactics (Lukasik et al. 2006). We ran a separate model for each measure of survival: (M5) annual survival, whether males survived until the next year, whereby a male that is never seen in all subsequent years in the trapping record is presumed dead = 0, and males found in the trapping record are recorded as survived = 1; (M6) on-site persistence continuous, the number of years a male was trapped as an adult, and (M7) on-site persistence binary, whether a male was trapped for more than one year, whereby one year = 0, and ≥ two years = 1). In models M6 and M7, we excluded all males that were captured in 2021 (as they were still living at the end of the study, and thus we cannot use on-site persistence as a proxy for lifespan). Docility and on-site persistence continuous were fitted as Poisson, and annual survival and on-site persistence binary were fitted as categorical responses. Capture and body condition were assigned as fixed effects. Tenure was also fitted as a fixed effect for annual survival models, however, for on-site persistence models (lifespan), tenure was excluded as a fixed effect because tenure is highly correlated to on-site persistence. Rainfall was also fitted as a fixed effect; for persistence on-site, annual rainfall prior to the field season was used (as in the fitness models above), however, for annual survival we used annual rainfall following the field season, as this variable represents the effect of rainfall on whether the male survived to the following field season. Tag, area and handler ID were fitted as random effects (Table 1).

Within- and among-individual covariance estimates were determined using the method described above, and we used non-informative parametric-expanded Wishart priors throughout all models, and ran all models for 4 000 000 iterations, with a burn-in of 5000 and thinning interval of 2000. Successive samples from the posterior distribution had low autocorrelation (the majority was *r* < 0.02, while all were *r* < 0.05). We standardized model covariance response variables (handling, tactic, and survival) to a scale from − 1 to 1 as described in Houslay and Wilson (2017a).

**Table 1:** Multivariate GLMM models examining the effect of docility (measured from 2014–2019) on survival and fitness of males at S.A. Lombard nature reserve. The following variables were included in all models: transfer, handing and reproductive tactic as covariate responses; capture, tenure, rainfall and body condition as fixed factors; tag, area and handler ID as random factors

Model	No. observations (n_males_)
M1 - Annual Offspring: Continuous	522(116)
M2- Annual Offspring: Binary	
M3 - Total Offspring: Continuous	198(42)
M4 - Total Offspring: Binary	
M5 – Annual Survival	638(181)
M6 - Persistence on-site: Continuous	683(156)
M7 - Persistence on-site: Binary	

## Results

### Variation in docility, reproductive output and survival

**Fig. 1:**
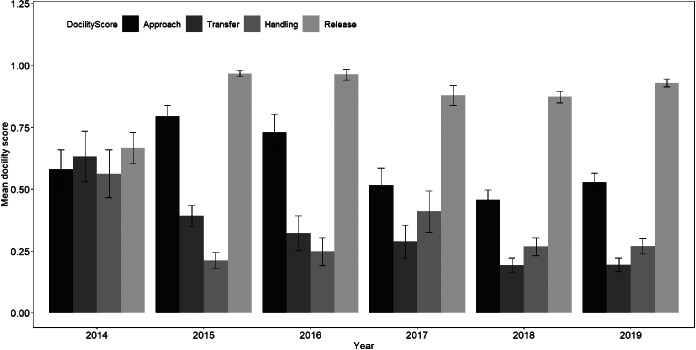
Mean ± SE docility score during approach, transfer, handling and release for all adult males sampled, by year

From 2014 until 2019, during the austral winter (May until August), we trapped, handled and scored docility 914 times for 274 male African ground squirrels. On average, males were sampled 3.3 times each (range 2–24 times per individual). Average docility varied from year to year (Fig. 1, Supplementary Table S1).

On an annual basis, most males (58%) had zero annual offspring, with few males siring more than one offspring (28% of males had one offspring, and 8%, 4%, 1% and 0.03% of males had 2,3,4 and 5 offspring, respectively; Supplementary Fig. S1, Supplementary Table S2). Over their lifetime, most males (79%) sired at least one offspring (Supplementary Fig. S2, Supplementary Table S3). Each male sired on average ± SE, 1.92 ± 0.18 offspring over their lifetime.

Annual survival ranged from 0.49 to 0.70 of the sampled male population (Supplementary Fig. S3, Supplementary Table S4). On-site persistence, a proxy for lifespan, ranged from 1 to 9 years. Approximately one third (0.34) of all males disappeared after the first year of adulthood with proportionally less males surviving subsequent years (on-site persistence: 1 year – 34% of males, 2 years – 24%, 3 years – 15%, 4 years – 11%, 5 years – 7%, 6 years – 5%, 7 years – 3%, 8 years – 1%. 9 years > 0.05%; Supplemental materials, Fig. S4, Table S5).

### Docility repeatability estimates

We examined repeatability of docility during approach, transfer, handling and release using 914 trapping occasions on 274 unique male African ground squirrels. We found moderate repeatability for transfer and handling, but we found no evidence of repeatability for approach and release (Table 2).

**Table 2:** Repeatability estimates for four models (M1-4) of docility behaviours measured during trapping as response variables. Fixed effects for each model included capture (first or subsequent capture), tenure, and rainfall. Random effects in each model included male ID (tag), capture area, and human handler ID. Significant results are bolded

Docility behaviour (response variable)	Repeatability						
	Link	R (SE)	95% CI	LRT full	D-stat	df	p
M1- Approach	Log	0 (0.02)	0–0.06	-830.7	-2.5 × 10^− 7^	1	1.000
M2- Transfer	**Log**	**0.31 (0.08)**	**0.11–0.41**	**-560.6**	**67.1**	**1**	**< 0.001**
M3 - Handling	**Log**	**0.31 (0.08)**	**0.12–0.43**	**-565.6**	**61.1**	**1**	**< 0.001**
M4 - Release	Logit	0.04 (0.09)	0–0.20	-244.1	3.48	1	0.031

### Effects of repeatable docility behaviours on reproductive fitness

**Fig. 2:**
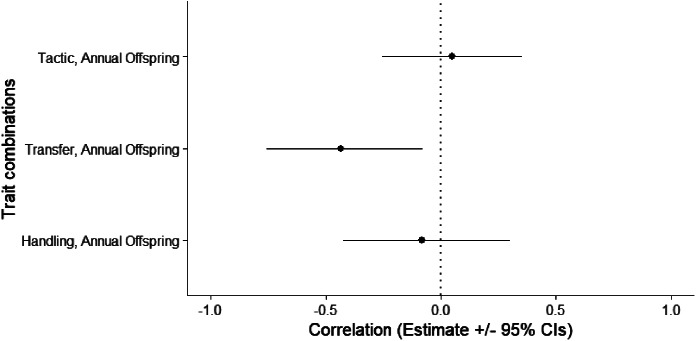
Correlations (estimate ± 95% credible intervals) between dependent variables (transfer, handling, tactic, annual fitness) derived from a MCMCglmm model multivariate model for docility behaviours during trapping. Individuals with low scores are more docile, and individuals with high scores are less docile. Significant results are defined as credible intervals that do not overlap with 0

**Fig. 3:**
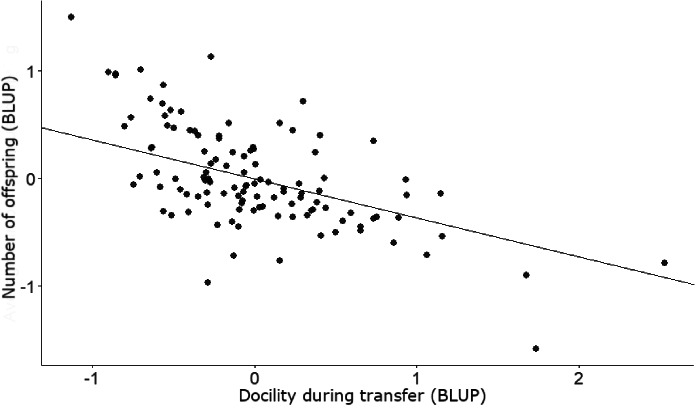
The association between docility during transfer and annual number of offspring, using posterior modes of random effects derived from the MCMCglmm multivariate model for docility behaviours (transfer, handling), tactic, and annual fitness. Each point represents a unique male. Individuals with low scores are more docile, and individuals with high scores are less docile. Note that for illustrative purposes (*sensu* Houslay and Wilson 2017b), for each individual we have plotted the best linear unbiased prediction (BLUP) value for docility and annual fitness (as we have multiple measures of docility and annual fitness per individual)

Out of the 274 males that were trapped and scored for docility, we had 116 unique males (522 observations) where an individual’s annual fitness had been determined and 42 unique males (198 observations) where we had determined each individual’s lifetime fitness, as well as reproductive tactic, tenure and body condition. We found some evidence of among-individual covariance between docility during transfer and annual fitness (transfer among-individual covariance = -0.43, CI = -0.76 to -0.08; Figs. 1 and 2; Table 3), and docility during transfer and annual fitness binary (transfer among-individual covariance = -0.35, CI = -0.67 to -0.01; Table 3). More docile males (lower scores) had more offspring than less docile males (Fig. 3). We found no evidence that docility affected lifetime fitness (Table 3).

We also found strong evidence that tenure affected annual offspring (annual offspring continuous β = 0.50, 95% CI = 0.34 to 0.66, pMCMC = < 0.0005; annual offspring binary β = 24.89, 95% CI = 1.47 to 43.05, pMCMC = < 0.0005); males with longer tenure (older) had more offspring. Body condition also affected annual offspring (annual offspring continuous β = 0.32, 95% CI = 0.17 to 0.47, pMCMC = 0.001; annual offspring binary β = 18.21, 95% CI = 0.89 to 30.95, pMCMC = < 0.0005); males in better body condition had more offspring (Table 4). We found that band males tended to be older and associated with higher rainfall (Supplementary Table S7).

**Table 3:** Among-individual correlations and HPD lower and upper credible intervals, between repeatable docility behavioural traits (transfer, handling), reproductive tactic (natal vs. band), fitness, and survival. The natal reproductive tactic is given as a reference, so the effect shown is that of the ‘band’ tactic. Significant correlations are bolded

Model	Correlated traits	Among-individual covariance	HPD Lower CI	HPD Upper CI
M1- Annual Offspring: Continuous	Handling, Annual Offspring	-0.08	-0.42	0.30
	**Transfer, Annual Offspring**	**-0.43**	**-0.76**	**-0.08**
	Band tactic, Annual Offspring	0.05	-0.26	0.35
M2 - Annual Offspring: Binary	Handling, Annual Offspring Binary	0.01	-0.33	0.39
	**Transfer, Annual Offspring Binary**	**-0.35**	**-0.67**	**-0.01**
	Band tactic, Annual Offspring Binary	-0.03	-0.30	0.28
M3 -Total Offspring: Continuous	Handling, Total Offspring	0.13	-0.28	0.53
	Transfer, Total Offspring	0.12	-0.26	0.52
	Band tactic, Total Offspring	-0.31	-0.62	0.09
M4 - Total Offspring: Binary	Handling, Lifetime Offspring Binary	0.14	-0.33	0.66
	Transfer, Lifetime Offspring Binary	-0.13	-0.56	0.34
	Band tactic, Lifetime Offspring Binary	-0.33	-0.73	0.11
M5 - Annual Survival	Handling, Annual Survival	-0.18	-0.54	0.12
	Transfer, Annual Survival	0.20	-0.11	0.50
	Band tactic, Annual Survival	0.07	-0.17	0.29
M6 – On-site Persistence: Continuous	Handling, On-site Persistence	0.01	-0.34	0.36
	Transfer, On-site Persistence	0.20	-0.13	0.54
	Band tactic, On-site Persistence	0.22	-0.02	0.44
M7 – On-site Persistence: Binary	Handling, On-site Persistence Binary	0.01	-0.34	0.36
	Transfer, On-site Persistence Binary	0.20	-0.13	0.54
	Band tactic, On-site Persistence Binary	0.22	-0.02	0.44

**Table 4:** Estimated means (β) and 95% credible intervals (95% CI) for tenure, rainfall and body condition (fixed factors) on fitness and survival variables. Effects of these fixed factors were also estimated for docility behaviours (transfer and handling) and are available in the Supplementary Table S7. Significant results are bolded

Model	Fixed effect	β	95% CI	pMCMC
M1- Annual Offspring: Continuous	**Tenure**	**0.5**	**0.34 – 0.66**	**< 0.0005**
	Rainfall	-0.1	-0.24 – 0.50	0.20
	**Body condition**	**0.32**	**0.17 – 0.47**	**0.001**
M2 - Annual Offspring: Binary	**Tenure**	**24.89**	**1.47 – 43.05**	**< 0.0005**
	Rainfall	-6.99	-16.59 – 0.24	0.06
	**Body condition**	**18.21**	**0.89 – 30.95**	**< 0.0005**
M3 - Total Offspring: Continuous	Tenure	0.06	-0.08 – 0.21	0.45
	Rainfall	-0.01	-0.10 – 0.08	0.78
	Body condition	-0.02	-0.11 – 0.09	0.78
M4 - Total Offspring: Binary	Tenure	0.19	-0.17 – 0.58	0.33
	Rainfall	0.03	-0.38 – 0.51	0.97
	Body condition	0.21	-0.34 – -0.81	0.15
M5 - Annual Survival	**Tenure**	**-66.63**	**-115.26 – -17.23**	**< 0.0005**
	**Rainfall**	**-9.02**	**-19.27 – -1.17**	**< 0.0005**
	Body condition	-3.49	-10.30 – 1.38	0.19
M6 - On-site Persistence: Continuous	Rainfall	0.004	-0.04 – 0.05	0.84
	Body condition	0.006	-0.06 – 0.06	0.88
M7 - On-site Persistence: Binary	Rainfall	0.03	-0.11 – 0.18	0.64
	Body condition	-0.16	-0.72 – 0.21	0.65

### Effects of repeatable docility behaviours on survival

Out of the 274 males scored for docility, we had 181 unique males (638 observations) where we had determined each individual’s annual survival, and 156 males (683 observations) where we had determined on-site persistence, as well as reproductive tactic, tenure and body condition. We consider tenure as a proxy for age, because the probability of ‘missing’ a male if it is within the trapping area is low. Of the 274 unique males in our study, we had 154 unique males that were first captured between 2011 and 2017, and found that 8% (13/154) of males were re-sighted after one year of being “missing” (un-trapped), while 3% (5/154) and 0.5% (1/154) of males were re-sighted after a gap of 2 and 3 years, respectively (Supplemental Materials Table S6). Males first trapped in 2018 and 2019, were excluded from this analysis, as we would not be able to determine if males unsighted after 2018, were resighted in the subsequent two years (2020 trapping data missing due to pandemic travel restrictions). On-site persistence ranged from 1 to 9 years; as males are sexually mature at approximately 9 months (Waterman 1996), males with 9 years of on-site persistence would have been ten years old at the youngest (if they were natal males, and older if they were band males, given that males disperse, on average, at an age of 3 years (O’Brien et al. 2021).

We found no evidence of among-individual covariance between docility (transfer and handling) and annual survival or on-site persistence (lifespan). Furthermore, we found no evidence that tactic and body condition influenced annual survival or on-site persistence (Table 4). However, in years of high rain, males had lower survival (annual survival continuous, β = -9.02, 95% CI = -19.27 to -1.17, pMCMC, 0.0005, Table 4). Furthermore, older males had lower annual survival (annual survival continuous, β = -35.65, 95% CI = -54.85 to -11.48, pMCMC, 0.0005, Table 4).

## Discussion

Our study examines the fitness consequences of between-individual variation in docility. We found strong evidence that males that were older, more docile and in better body condition had higher annual reproductive fitness, suggesting that from year-to-year docility contributes to, or is related to traits that influence reproductive skew in Cape ground squirrels (Manjerovic et al. 2022). However, higher docility did not increase reproductive output over an individual’s lifetime. Our findings suggest that any benefits and costs of docility for reproduction may be interacting with ecological factors that vary from year to year, such that overall, any given docility phenotype is not advantageous over a longer period of time. Furthermore, we found no evidence that docility affected annual survival or on-site persistence (lifespan), suggesting that between-individual variation in docility may not affect group-living benefits such as enhanced predator detection and deterrence (Waterman 1997; Edwards and Waterman 2011).

Our finding of a lack of relationship between docility personality types and survival and lifetime reproduction does not shed light on why Cape ground squirrels have docile personality types (Warrington et al. 2022). However, variation in personality traits can arise via several proximal mechanisms including genetic heritability and developmental variation, such as differences in early-life conditions (Réale and Montiglio 2021). Furthermore, personality traits may be driven by many ecological factors, and therefore benefits associated with docility may be highly contextualized (e.g., Haines et al. 2020).

### Docility and reproductive output

More docile males tended to have higher annual reproductive output than less docile males. This finding is consistent with other studies that found that personality traits affected reproductive success (e.g., Boon et al. 2007; Ariyomo and Watt 2012; Betini and Norris 2012; Le Cœur et al. 2015). Our findings suggest that on an annual basis, docility may contribute to reproductive skew in males via several potential mechanisms, although mechanisms remain to be tested.

One such mechanism that may drive an association between personality traits and reproductive success is mate choice. As personality can be an honest signal of male quality, females may use personality traits to select mates (Schuett et al. 2010). For example, in Trinidadian guppies, *Poecilia reticulata*, females preferred bold males (Godin and Dugatkin 1996). In Cape ground squirrels, an estrous female will mate on average with 4 males during her oestrus period, even though the operational sex ratio is 11 males to 1 female (Waterman 1996, 1998, 2010). Reproductive skew is high with only approximately 1/3 of males siring offspring (Manjerovic and Waterman 2015) suggesting that female choice may be influencing male reproductive success (Schuett et al. 2010). Perhaps females prefer more docile males.

However, given that docility may form a behavioural syndrome with other traits (Sih et al. 2004), docility may not actually be directly selected for by females, but instead may be associated with different traits influenced by female choice (Schuett et al. 2010). Perhaps, in Cape ground squirrels, females may not be choosing mates based on docility, but may be choosing traits associated with docility (that we have not measured in this study). Alternatively, females may be selecting mates by assessing multiple traits (Clutton-Brock and McAuliffe 2009). For example, we found that older males also had higher annual reproductive success, suggesting that females also prefer older males. This result is consistent with a different study of a Namibian population of Cape ground squirrels where younger subordinate males were rejected by the female (Waterman 1998). Furthermore, older Cape ground squirrel males tend to be in better body condition (O’Brien et al. 2021). In fact, our findings show that males with better body condition had higher fitness, as in other species (e.g., Preston et al. 2003). However, as docility is not associated with body condition in Cape ground squirrel males (Warrington et al. 2022), and inversely related to age (older males are less docile), the apparent female choice for docility is likely not a by-product of a choice for better body condition, or driven by a female preference for older males. Clearly, many factors other than behavioural traits influence reproductive success in males.

Consequently, personality traits may vary in their relative reproductive success from year to year, and thus, can be highly contextualized (e.g., Réale et al. 2009; Haines et al. 2020). We found that males that were consistently more docile did not have higher lifetime reproductive fitness, suggesting that the advantages of docility are not consistent from year to year. Perhaps the benefits/costs of docility may be interacting with ecological factors that vary annually, such that overall, any given docility phenotype is not advantageous over the course of a lifetime. Indeed, the fitness advantages of particular categories of personalities are highly dependent on environmental factors such as the social environment (Montiglio et al. 2017) and resource availability (Haines et al. 2020). For example, the fitness benefit of docility depends on age in bighorn sheep rams, *Ovis canadensis*, with docility being positively associated with fitness in older, but not necessarily, younger rams (Réale et al. 2009). Also, in North American red squirrels, *Tamiasciurus hudsonicus*, the benefits of aggressive personalities depend on whether a male experienced a mast (resource pulse) year. For males that had experienced a mast year, the more aggressive males had higher fitness, while for males that had not experienced a mast year, less aggressive males had higher fitness (Haines et al. 2020). In Cape ground squirrels, older males had higher reproductive success, although older males are on average less docile (this study, Warrington et al. 2022), demonstrating that both age and personality type influence reproductive success.

### Docility and survival

Males that form groups may benefit by having increased survival as a consequence of group-enhanced antipredator behaviours (Silk 2007). Male Cape ground squirrels group largely as a result of the benefits of enhanced predator detection and deterrence (Waterman 1997; Edwards and Waterman 2011), and lone males spend more time vigilant and less time foraging than those in groups (Scantlebury et al. 2008). However, we found no evidence that docility influenced survival rates. Similarly, personalities were not associated with survival in the North American red squirrels, *T. hudsonicus* (active and aggressive personalities; Haines et al. 2020), which may indicate that in some species consistency in behavioural traits may not be beneficial for survival.

Additionally, both annual survival and on-site persistence (lifespan), were unrelated to body condition, which is surprising because body condition tends to be related to survival in other small mammals (Bright Ross et al. 2021). Furthermore, annual survival was negatively influenced by rainfall, in that higher rainfall was associated with low survival probability the following year. This was initially surprising, given that rainfall is related to plant productivity, a crucial food source for Cape ground squirrels (Herzig-Straschil 1978). However, rainfall patterns are highly variable at our field site and fluctuate from year to year (O’Brien et al. 2021; Manjerovic et al. 2022), and the link between survival and rainfall/body condition is complex because foraging efficiency likely varies between males of different ages and reproductive tactics (Scantlebury et al. 2008). Furthermore, at our study site, years of higher rainfall may also have been associated with days of intense rainfall, with localized flooding (unpublished data). Thus, future studies need to considerate different measures of rainfall, such as rainfall variability and maximum daily rainfall amounts. Nonetheless, the benefits of docility may influence survival in nuanced ways with benefits depending on contexts such as tactic, age, and environmental conditions. Indeed, other studies have shown that the benefits of personalities are highly context dependent (e.g., Montiglio et al. 2017; Wauters et al. 2021).

Also, given that behavioural traits may influence social group features (Webster and Ward 2011), variations in the docility of group members may influence the costs and benefits of group membership. For example, docility may influence social behaviours such as affiliative behaviours (e.g., grooming) or agonistic behaviours (e.g., aggression), which consequently may influence access to the important social or health benefits provided by receiving affiliative behaviours, which may have downstream effects on survival. Indeed, in primates, male-male associations provide benefits such as cooperative group defense, dispersal partners, ectoparasite removal, and thermoregulation (Jack and Riley 2014).

### What might be the benefits of docility in Cape ground squirrels?

There are several features associated with Cape ground squirrels that may be benefited by variation in docility. First, Cape ground squirrels are long-lived (10 years, this study), and older males have higher reproductive success. One third of the males in our study disappeared (majority presumed dead) after 1 year of adulthood had passed (Fig. S4), but of those that survived, most produced at least one offspring over their lifetime (79% of males, Fig. S2, Table S3). This suggests that surviving long enough to reproduce is important to male Cape ground squirrels (we note that a more comprehensive study examining all the key factors influencing survival and lifetime fitness is needed, and is not the focus of this study). Given that Cape ground squirrels live in groups, and grouping has largely been attributed to antipredator benefits (Waterman 1997), efficient social functioning may be facilitated by behaviour variations amongst individuals (Bergmüller and Taborsky 2010). Thus, behavioural variation, such as docility, may help Cape ground squirrel males to survive long enough to reproduce, and successfully navigate the high reproductive competition associated with the high male reproductive skew (Fig. S1, Table S2; Manjerovic and Waterman 2015).

Consequently, cooperation among males seems to be an important strategy in Cape ground squirrels. Therefore, between-individual variation in docility may also be beneficial if docility enhances the benefits of social living and cooperation in Cape ground squirrels. All-male groups are rare, but can be found in several species from different lineages (e.g., primates, Strier 1994; otters, Blundell et al. 2004; rodents, van der Marel et al. 2020; dolphins, Connor et al. 2022). In these species, selection may have occurred for behaviours that limit social intolerance (DeTroy et al. 2022). If cooperation leads to higher fitness (Silk 2007), we would then expect male fitness to be positively associated with behavioural traits (such as high docility), which may enhance cooperation and grouping among males (Anderson 2007; Hare et al. 2007). However, docility may indirectly affect fitness by influencing other social features such as social interaction and networks. Accordingly, further studies examining the effect of docility on social dynamics would elucidate the role that docility personalities play in this species.

### Study limitations

The survival and fitness consequences for differing personality traits may also vary according to environmental conditions such as predation pressure (Réale and Festa-Bianchet 2003), food availability (Dingemanse et al. 2004; Le Cœur et al. 2015), social condition (Both et al. 2005), and anthropogenic disturbances (Brehm et al. 2019). Therefore, the fitness of different behavioural phenotypes may be equal on average across a landscape of differing conditions (Boon et al. 2007), even if individuals with different personality traits have advantages in some areas/conditions of their habitat over others (Brehm et al. 2019). Indeed, male Cape ground squirrels vary in the size of their home range (Waterman 1995), feed on a variety of food resources (Herzig-Straschil 1978), inhabit areas with varying degrees of predation and human disturbance (Unck et al. 2009), and demonstrate variation and fluidity in their social networks (fission-fusion society, Manjerovic and Waterman 2015). Consequently, Cape ground squirrels live within a landscape of varying ecological (environmental and social) conditions, such that this study may have failed to fully capture the effects of differing personality types (docile versus non-docile) on survival. Accordingly, further studies examining the role of ecological heterogeneity in influencing behaviours may give greater insight into the role docility plays in the fitness and survival of this species.

Furthermore, context-dependent benefits of docility might imply that there are benefits to being behaviourally plastic. In fact, it is possible for an individual’s behaviour to be both consistently different between individuals and exhibit a level of individual plasticity (Dingemanse et al. 2010; Montiglio et al. 2017). However, our study did not estimate within- individual plasticity of docility behaviours. Yet, in Cape ground squirrels, there is potential for differing benefits of docility, and thus potential benefits to plasticity in docility, because males exhibit two discrete alternative reproductive tactics (Scantlebury et al. 2008), with the benefits of each tactic interacting with environmental factors (O’Brien et al. 2018).

Thus, considering the variation of life history features and social associates (e.g., sex, age, breeding status) among males, we would expect that a male’s behaviour may influence the extent and types of interactions they have with other group members, which may have downstream consequences to reproductive success and survival. Likewise, we might expect that the benefits of docility will depend on context, and thus, further studies investigating interactions between docility, and plasticity on docility, might contribute to how variation in docility is maintained in this species. Furthermore, the apparent large variation in environmental conditions (e.g., rainfall, temperature) seen at our field site (Warrington and Waterman 2022), in conjunction with the variation in social groupings and attributes seen in Cape ground squirrels (Waterman 1995, 2002; Skurski and Waterman 2005), may suggest that ecological variation plays a role in maintaining individual docility variation in this species.

Moreover, variation in behaviours may be maintained via many diverse proximal mechanisms, including internal (e.g., genetic (heritability), physiological), external (e.g., environmental and social conditions) and the interaction between multiple drivers (Wolf and Weissing 2010). Additionally, the relative contribution of drivers to behavioural variation may vary with time, such as across different ages or life stages (Réale and Montiglio 2021). Further studies examining proximal mechanisms that may influence docility variation would aid in understanding the role docility plays in this low aggression species.

## Conclusion

There are different ways that individuals could maximize their fitness. However, within species, each individual has different constraints on the strategies they can use. Different individuals may employ different behavioural strategies (Biro and Stamps 2008). Species where males persist in multi-male groups are rare, (e.g., Strier et al. 1994; Blundell et al. 2004; van der Marel et al. 2020; Connor et al. 2022) and these species may represent an alternate route to the evolution of male sociality, such that examining the effect of behavioural traits on fitness is valuable. Our finding that low docility was related to annual reproductive output, but not lifetime fitness, indicates that high docility may be beneficial and perhaps variation in docility may be related to social living or all-male groupings in this group-living species. As the study of animal behaviour has been biased in the past towards taking the perspective of aggression and conflict (Griffith 2019), studying these non-aggressive societies may increase our understanding of the different drivers of male sociality and the role behavioural traits play in the evolution of sociality and cooperation.

### Electronic supplementary material

Below is the link to the electronic supplementary material.

Supplementary Material 1

Supplementary Material 2

## Data Availability

All data analyzed for this study are included in this published article and its supplementary information files.
