# Supplementary material for: Lovers, not fighters: docility influences reproductive fitness, but not survival, in male Cape ground squirrels, Xerus inauris
Source: Behav Ecol Sociobiol. 2024 Jan 4;78(1):6. doi: 10.1007/s00265-023-03421-8 (PMC10766660; doi:10.1007/s00265-023-03421-8)
Supplement: Supplementary file 1 — Supplementary Material 1 [file 265_2023_3421_MOESM1_ESM.docx]

**Supplementary Materials**

Lovers, not fighters: Docility influences reproductive fitness, but not survival, in male Cape ground squirrels, *Xerus inauris*

Journal: Behavioral Ecology and Sociobiology

Miyako H Warrington^a,b*^, Sienna Beaulieu^b^, Riley Jellicoe^b^, Sjoerd Vos^b,c^, Nigel C Bennett^d^, Jane M Waterman^b,d^

1. Department of Biological and Medical Sciences, Oxford Brookes University, Oxford, UK
2. Department of Biological Sciences, University of Manitoba, Winnipeg, MB, Canada
3. Graduate School of Life Sciences, University of Utrecht, Utrecht, Netherlands
4. Mammal Research Institute, Department of Zoology and Entomology, University of Pretoria, Pretoria 0002, South Africa

*Corresponding author: Miyako H Warrington, mwarrington@brookes.ac.uk

**Descriptive statistics for docility**

Table S1: Mean ± SE docility score during approach, transfer, handling and release for all adult males sampled, by year. Males were sampled 914 times over the years, and this represented 274 unique males that were sampled on average 3.3 times each (range 2-24 times per individual).

|  |  |  | Mean±SE docility score | | | |
| --- | --- | --- | --- | --- | --- | --- |
| Year | N sample size | N unique males | Approach | Transfer | Handling | Release |
| 2014 | 55 | 24 | 0.58±0.08 | 0.63±0.10 | 0.56±0.10 | 0.67±0.06 |
| 2015 | 227* | 80* | 0.79±0.05 | 0.39±0.04 | 0.22±0.03 | 0.97±0.01 |
| 2016 | 83* | 73* | 0.73±0.07 | 0.32±0.07 | 0.25±0.06 | 0.96±0.02 |
| 2017 | 66 | 61 | 0.52±0.07 | 0.28±0.07 | 0.41±0.08 | 0.88±0.04 |
| 2018 | 216* | 119* | 0.46±0.04 | 0.19±0.03 | 0.27±0.04 | 0.87±0.02 |
| 2019 | 267* | 125* | 0.53±0.04 | 0.19±0.03 | 0.27±0.03 | 0.93±0.02 |
| 2014-2019 | 914 | 274 |  |  |  |  |

* N sample size (N unique males) for release in N2015 = 209(73), N2016 = 82(72), N2018 = 214,(117), N2019 = 266(124)

**Descriptive statistics for reproductive output**

**
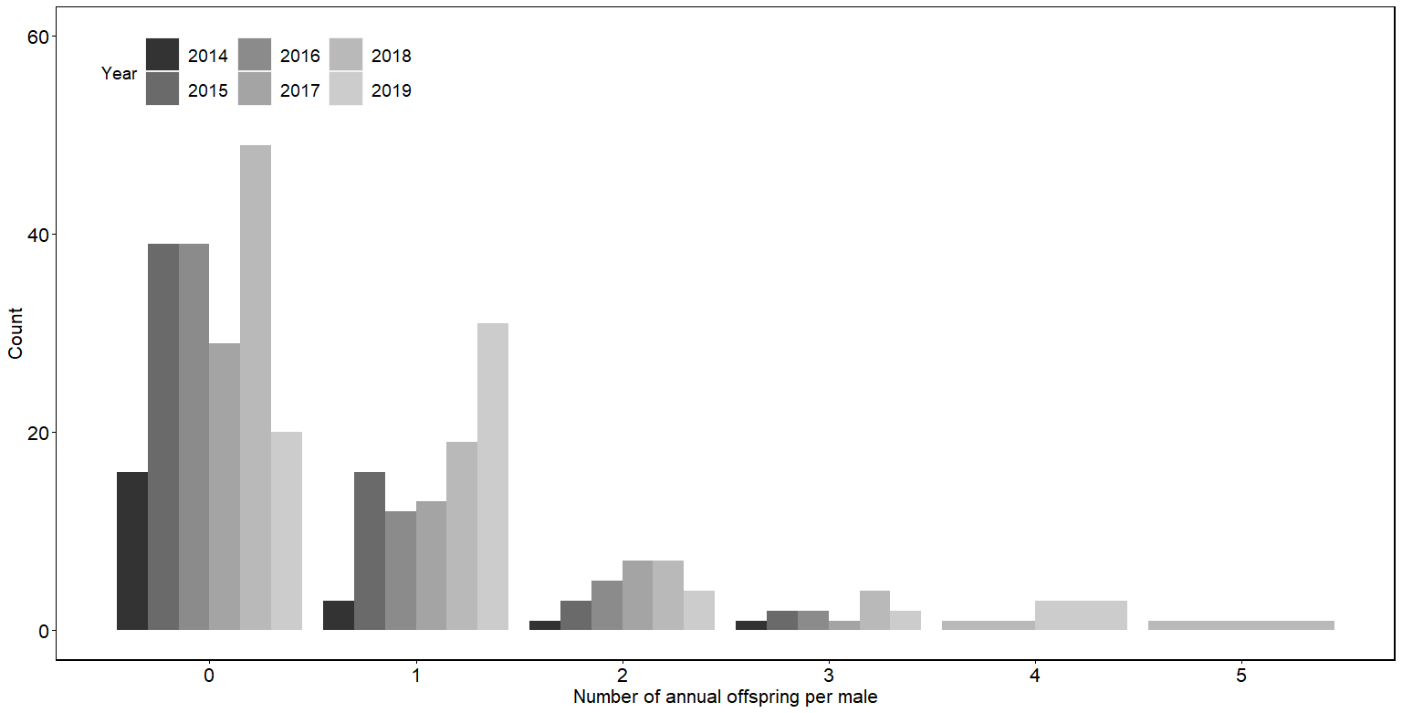
**

Fig.S1: Histogram of the number of annual offspring for each unique male identification by year (unique TagYear).

**
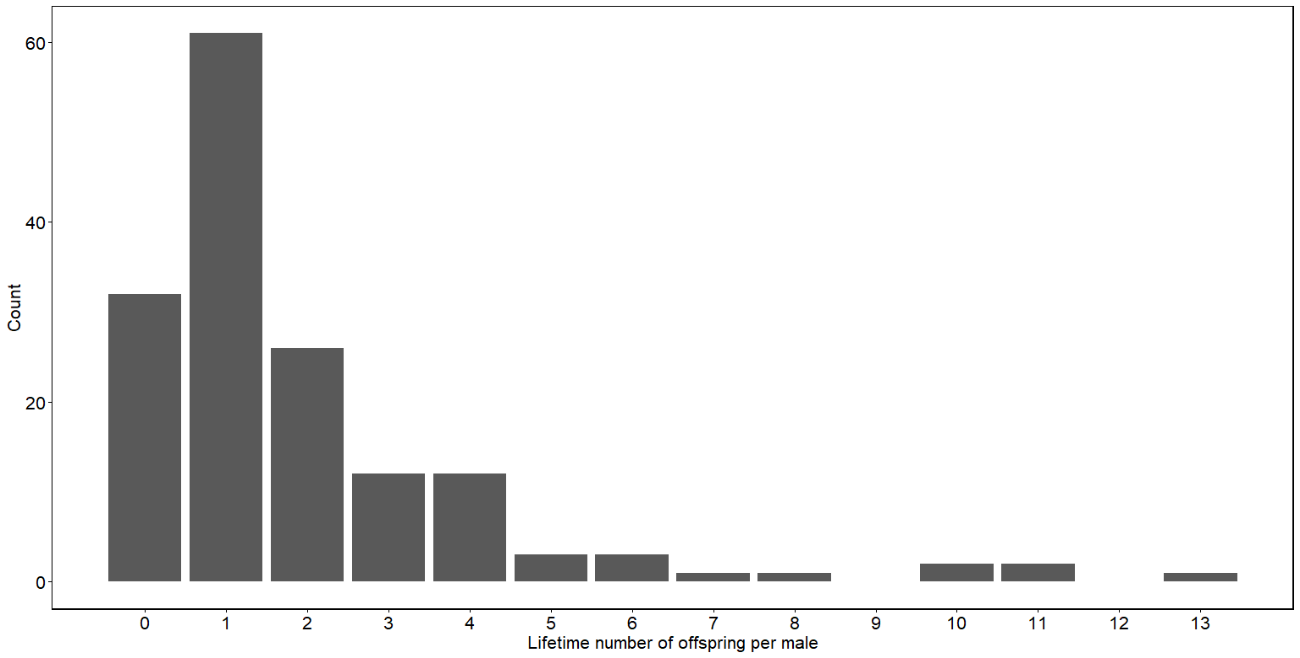
**

Fig.S2: Histogram of the total number of offspring over their lifetime for each unique male identification (unique Tag ID).

Table S2: Mean ± SE number of annual offspring for each unique male identification by year (unique TagID-Year).

|  | Number of males | | | | | |
| --- | --- | --- | --- | --- | --- | --- |
| Year | Zero offspring | 1 offspring | 2 offspring | 3 offspring | 4 offspring | 5 offspring |
| 2014 | 16 | 3 | 1 | 1 | 0 | 0 |
| 2015 | 39 | 16 | 3 | 2 | 0 | 0 |
| 2016 | 39 | 12 | 5 | 2 | 0 | 0 |
| 2017 | 29 | 13 | 7 | 1 | 0 | 0 |
| 2018 | 49 | 19 | 7 | 4 | 1 | 1 |
| 2019 | 20 | 31 | 4 | 2 | 3 | 0 |
| Total | 192 | 94 | 27 | 12 | 4 | 1 |
| Prop. | 0.58 | 0.28 | 0.08 | 0.04 | 0.01 | 0.003 |

Table S3: Lifetime reproductive success (measured by the total number of offspring) for each unique male identification (unique Tag ID). For N = 120 males, we could not determine lifetime reproductive success because they were still alive at the end of the study (in 2019).

| Total # offspring | # unique males |
| --- | --- |
| 0 | 32 |
| 1 | 61 |
| 2 | 26 |
| 3 | 12 |
| 4 | 12 |
| 5 | 3 |
| 6 | 3 |
| 7 | 1 |
| 8 | 1 |
| 10 | 2 |
| 11 | 2 |
| 13 | 1 |

**Descriptive statistics for survival**

**
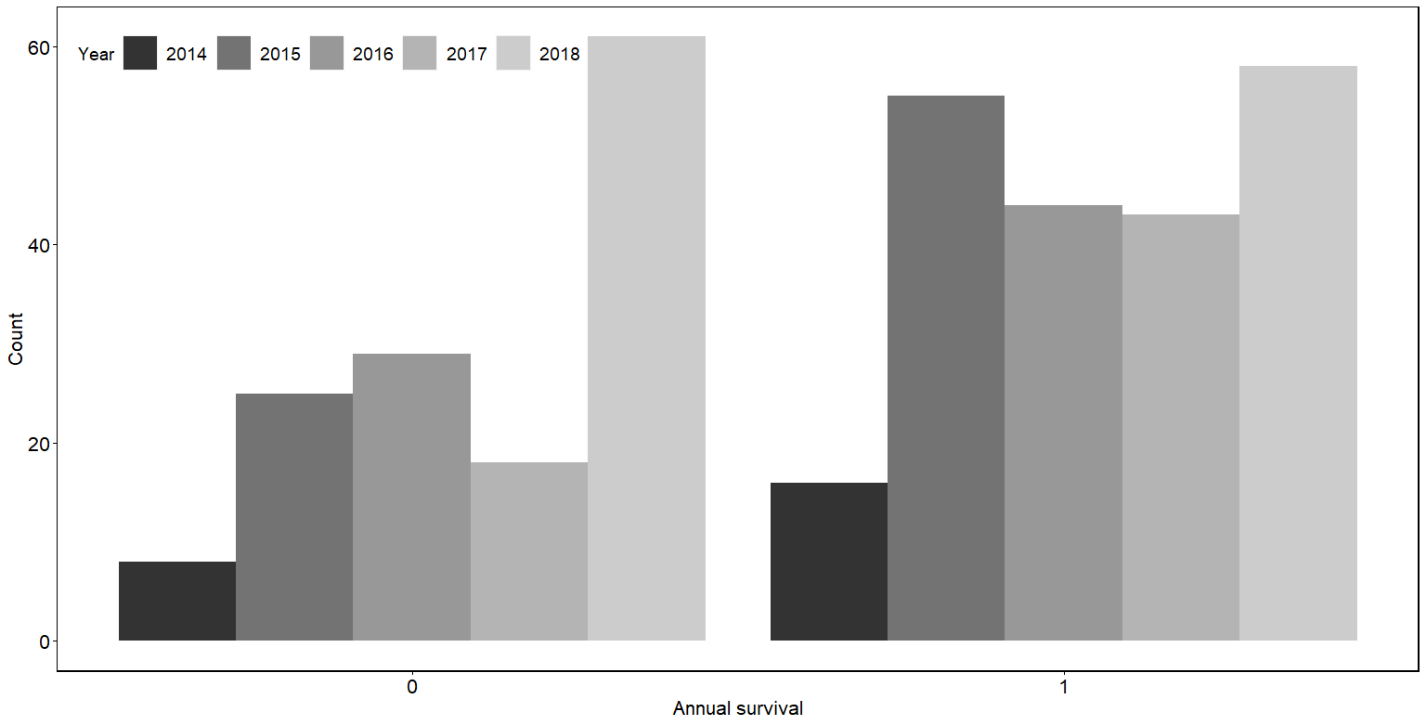
**

Fig. S3: Histogram of the annual survival ( 0 = disappeared/ not re-sighted; 1= trapped or observed) the followed year for each unique male identification per year (unique TagYear).


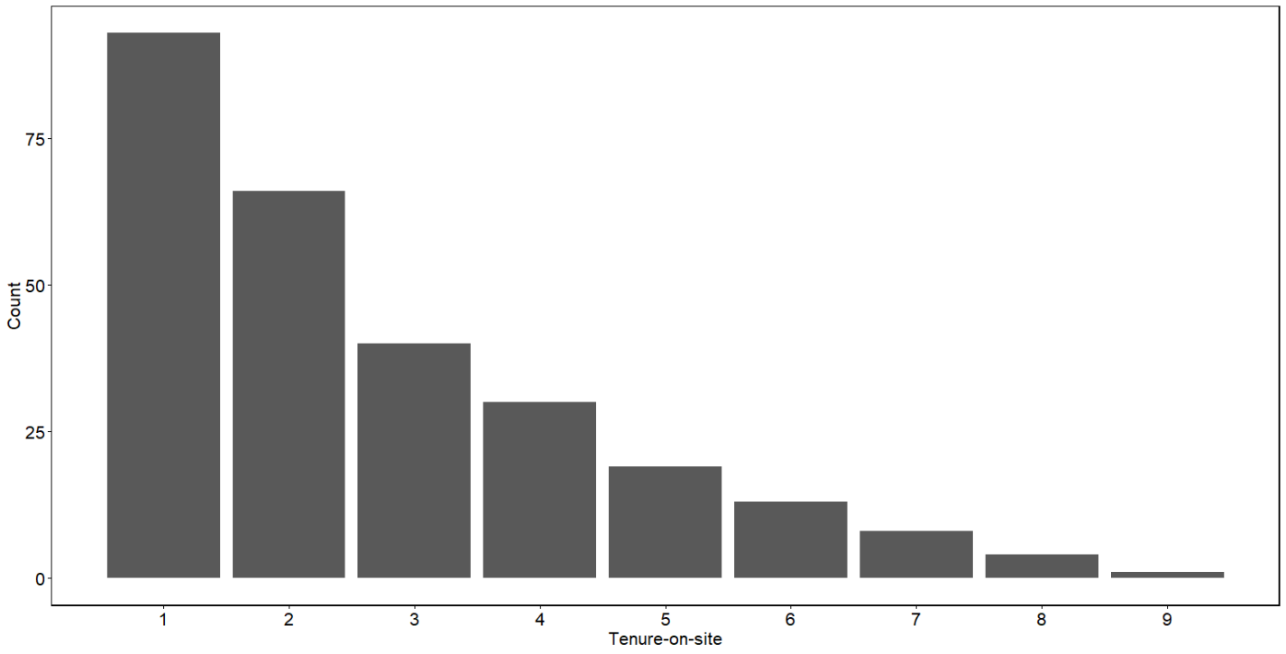


Fig. S4: On-site persistence, a proxy for lifespan for males (N=274) captured and scored for docility at S.A. Lombard Nature reserve from 2014-2015.

Table S4: The number and proportion of males (unique TagYears) that were re-sighted (survived) the following year.

| Year | Number disappeared | Number survived | Prop survival |
| --- | --- | --- | --- |
| 2014 | 8 | 16 | 0.67 |
| 2015 | 25 | 55 | 0.69 |
| 2016 | 29 | 44 | 0.60 |
| 2017 | 18 | 43 | 0.70 |
| 2018 | 61 | 58 | 0.49 |

Table S5: On-site persistence, a proxy for lifespan for males (N=236) captured and scored for docility at S.A. Lombard nature reserve from 2011-2015.

| Tenure on-site | # unique males | Prop of males |
| --- | --- | --- |
| 1 | 93 | 0.34 |
| 2 | 66 | 0.24 |
| 3 | 40 | 0.15 |
| 4 | 30 | 0.11 |
| 5 | 19 | 0.07 |
| 6 | 13 | 0.05 |
| 7 | 8 | 0.03 |
| 8 | 4 | 0.01 |
| 9 | 1 | 0.004 |

**Capture history**

Table S6: Gaps in capture history of males (N=154) first captured from 2011-2017 at S.A.Lombard nature reserve. Males first captured in 2018 and 2019 (N=121) were excluded as 2-3 years of continuous subsequent capture history was unavailable. Note that travel restriction in 2020 due to the COVID-19 pandemic restrictions created a gap in the trapping records at our study site.

|  | Skipped trapping | | | |  |
| --- | --- | --- | --- | --- | --- |
| First year trapped | 0 years | 1 year | 2 years | 3 years | Total Males |
| 2011 | 8 | 0 | 1 | 0 | 9 |
| 2012 | 6 | 0 | 0 | 0 | 6 |
| 2013 | 24 | 3 | 1 | 1 | 28 |
| 2014 | 14 | 1 | 0 | 0 | 15 |
| 2015 | 32 | 3 | 2 | 0 | 37 |
| 2016 | 22 | 2 | 0 | 0 | 24 |
| 2017 | 30 | 4 | 1 | 0 | 35 |
| 2011-2017 | 136 | 13 | 5 | 1 | 154 |
| Proportion | 0.88 | 0.08 | 0.03 | 0.006 |  |

**Effect of fixed factors**

Table S7: The effect of fixed factors on all response covariates (repeatable docility measures (transfer, handling), reproductive tactic, and fitness/survival measures). The natal reproductive tactic is given as a reference, so the effect shown is that of the ‘band’ tactic. Significant effects are bolded.

| Model | Fixed effect | Response variable | β | 95% CI | pMCMC |
| --- | --- | --- | --- | --- | --- |
| M1- Annual Offspring: Continuous | **Tenure** | **Annual Offspring: Continuous** | **0.5** | **0.34 ̶ 0.66** | **<0.0005** |
|  | Tenure | Docility: transfer | -0.17 | -0.44 ̶ 0.12 | 0.29 |
|  | Tenure | Docility: handling | 0.17 | -0.09 ̶ 0.40 | 0.19 |
|  | **Tenure** | **Band tactic** | **60.16** | **29.05 ̶ 100.17** | **<0.0005** |
|  | Rainfall | Annual Offspring: Continuous | -0.1 | -0.24 ̶ 0.50 | 0.20 |
|  | Rainfall | Docility: transfer | -0.15 | -0.36 ̶ 0.09 | 0.2 |
|  | Rainfall | Docility: handling | 0.04 | -0.19 ̶ 0.28 | 0.76 |
|  | **Rainfall** | **Band tactic** | **9.233** | **1.31 ̶ 16.67** | **0.004** |
|  | **Body condition** | **Annual Offspring: Continuous** | **0.32** | **0.17 ̶ 0.47** | **0.001** |
|  | Body condition | Docility: transfer | -0.08 | -0.31 ̶ 0.15 | 0.49 |
|  | Body condition | Docility: handling | -0.07 | -0.31 ̶ 0.14 | 0.46 |
|  | Body condition | Band tactic | 2.88 | -5.08 ̶ 11.90 | 0.51 |
| M2 - Annual Offspring: Binary | **Tenure** | **Annual Offspring: Binary** | **24.89** | **1.47 ̶ 43.05** | **<0.0005** |
|  | Tenure | Docility: transfer | -0.17 | -0.46 ̶ 0.12 | 0.27 |
|  | Tenure | Docility: handling | 0.17 | -0.08 ̶ 0.41 | 0.20 |
|  | **Tenure** | **Band tactic** | **65.87** | **26.95 ̶ 97.74** | **<0.0005** |
|  | Rainfall | Annual Offspring: Binary | -6.99 | -16.59 ̶ 0.24 | 0.06 |
|  | Rainfall | Docility: transfer | -0.15 | -0.38 ̶ 0.08 | 0.21 |
|  | Rainfall | Docility: handling | 0.05 | -0.18 ̶ 0.28 | 0.71 |
|  | **Rainfall** | **Band tactic** | **9.88** | **0.18 ̶ 29.66** | **0.002** |
|  | **Body condition** | **Annual Offspring: Binary** | **18.21** | **0.89 ̶ 30.95** | **<0.0005** |
|  | Body condition | Docility: transfer | -0.11 | -0.35 ̶ 0.11 | 0.36 |
|  | Body condition | Docility: handling | -0.09 | -0.33 ̶ 0.12 | 0.40 |
|  | Body condition | Band tactic | 3.91 | -6.15 ̶ 14.88 | 0.39 |
| M3 - Total Offspring: Continuous | Tenure | Total Offspring: Continuous | 0.06 | -0.08 ̶ 0.21 | 0.45 |
|  | Tenure | Docility: transfer | -0.41 | -0.97 ̶ 0.18 | 0.14 |
|  | Tenure | Docility: handling | -0.15 | -0.69 ̶ 0.32 | 0.57 |
|  | **Tenure** | **Band tactic** | **14.27** | **5.66 ̶ 23.39** | **<0.0005** |
|  | Rainfall | Total Offspring: Continuous | -0.01 | -0.10 ̶ 0.08 | 0.78 |
|  | Rainfall | Docility: transfer | -0.2 | -0.59 ̶ 0.18 | 0.3 |
|  | Rainfall | Docility: handling | -0.07 | -0.42 ̶ 0.31 | 0.68 |
|  | Rainfall | Band tactic | 1.78 | -0.26 ̶ 4.07 | 0.1 |
|  | Body condition | Total Offspring: Continuous | -0.02 | -0.11 ̶ 0.09 | 0.78 |
|  | Body condition | Docility: transfer | 0.11 | -0.24 ̶ 0.51 | 0.57 |
|  | Body condition | Docility: handling | 0.07 | -0.28 ̶ 0.39 | 0.66 |
|  | Body condition | Band tactic | 0.34 | -2.15 ̶ 3.16 | 0.84 |
| M4 - Total Offspring: Binary | Tenure | Total Offspring: Binary | 0.19 | -0.17 ̶ 0.58 | 0.33 |
|  | Tenure | Docility: transfer | -0.32 | -0.86 ̶ 0.23 | 0.25 |
|  | Tenure | Docility: handling | -0.14 | -0.63 ̶ 0.35 | 0.57 |
|  | **Tenure** | **Band tactic** | **14.17** | **3.53 ̶ 21.87** | **<0.0005** |
|  | Rainfall | Total Offspring: Binary | 0.03 | -0.38 ̶ 0.51 | 0.97 |
|  | Rainfall | Docility: transfer | -0.22 | -0.60 ̶ 0.15 | 0.23 |
|  | Rainfall | Docility: handling | -0.04 | -0.42 ̶ 0.28 | 0.78 |
|  | **Rainfall** | **Band tactic** | **3.18** | **0.49 ̶ 6.76** | **0.02** |
|  | Body condition | Total Offspring: Binary | 0.21 | -0.34 ̶ -0.81 | 0.15 |
|  | Body condition | Docility: transfer | 0.08 | -0.29 ̶ 0.48 | 0.72 |
|  | Body condition | Docility: handling | 0.07 | -0.28 ̶ 0.42 | 0.73 |
|  | Body condition | Band tactic | -1.08 | -3.96 ̶ 1.56 | 0.48 |
| M5 - Annual Survival | **Tenure** | **Annual Survival** | **-66.63** | **-115.26 ̶ -17.23** | **<0.0005** |
|  | Tenure | Docility: transfer | -0.22 | -0.51 ̶ 0.06 | 0.13 |
|  | Tenure | Docility: handling | 0.21 | -0.03 ̶ 0.46 | 0.11 |
|  | **Tenure** | **Band tactic** | **43.63** | **16.28 ̶ 70.33** | **<0.0005** |
|  | **Rainfall** | **Annual Survival** | **-9.02** | **-19.27 ̶ -1.17** | **<0.0005** |
|  | Rainfall | Docility: transfer | -0.14 | -0.34 ̶ 0.05 | 0.14 |
|  | Rainfall | Docility: handling | 0.06 | -0.13 ̶ 0.25 | 0.58 |
|  | **Rainfall** | **Band tactic** | **8.63** | **1.69 ̶ 15.53** | **0.001** |
|  | Body condition | Annual Survival | -3.49 | -10.30 ̶ 1.38 | 0.19 |
|  | Body condition | Docility: transfer | -0.07 | -0.27 ̶ 0.12 | 0.46 |
|  | Body condition | Docility: handling | -0.08 | -0.26 ̶ 0.12 | 0.42 |
|  | Body condition | Band tactic | 0.69 | -6.65 ̶ 7.80 | 0.85 |
| M6 - On-site Persistence : Continuous | Tenure | Docility: transfer | -0.18 | -0.49 ̶ 0.14 | 0.25 |
|  | Tenure | Docility: handling | 0.11 | -0.14 ̶ 0.37 | 0.42 |
|  | **Tenure** | **Band tactic** | **19.41** | **9.28 ̶ 28.31** | **<0.0005** |
|  | Rainfall | On-site Persistence : Continuous | 0.004 | -0.04 ̶ 0.05 | 0.84 |
|  | Rainfall | Docility: transfer | -0.13 | -0.35 ̶ 0.07 | 0.21 |
|  | Rainfall | Docility: handling | 0.04 | -0.18 ̶ 0.26 | 0.75 |
|  | **Rainfall** | **Band tactic** | **4.88** | **1.13 ̶ 7.96** | **<0.0005** |
|  | Body condition | On-site Persistence : Continuous | 0.006 | -0.06 ̶ 0.06 | 0.88 |
|  | Body condition | Docility: transfer | -0.07 | -0.27 ̶ 0.15 | 0.47 |
|  | Body condition | Docility: handling | -0.06 | -0.28 ̶ 0.14 | 0.61 |
|  | Body condition | Band tactic | -0.88 | -4.10 ̶ 2.03 | 0.61 |
| M7 - On-site Persistence: Binary | Tenure | Docility: transfer | -0.1 | -0.38 ̶ 0.17 | 0.52 |
|  | Tenure | Docility: handling | 0.11 | -0.13 ̶ 0.34 | 0.38 |
|  | **Tenure** | **Band tactic** | **12.36** | **4.32 ̶ 18.09** | **<0.0005** |
|  | Rainfall | On-site Persistence: Binary | 0.03 | -0.11 ̶ 0.18 | 0.64 |
|  | Rainfall | Docility: transfer | -0.15 | -0.34 ̶ 0.05 | 0.13 |
|  | Rainfall | Docility: handling | 0.04 | -0.18 ̶ 0.27 | 0.76 |
|  | **Rainfall** | **Band tactic** | **2.88** | **0.65 ̶ 4.67** | **<0.0005** |
|  | Body condition | On-site Persistence: Binary | -0.16 | -0.72 ̶ 0.21 | 0.65 |
|  | Body condition | Docility: transfer | -0.07 | -0.31 ̶ 0.10 | 0.48 |
|  | Body condition | Docility: handling | -0.04 | -0.25 ̶ 0.16 | 0.67 |
|  | Body condition | Band tactic | -0.23 | -2.19 ̶ 1.62 | 0.83 |
